# Multicriteria Decision Making Method Based on the Higher Order Hesitant Fuzzy Soft Set

**DOI:** 10.1155/2014/873454

**Published:** 2014-08-21

**Authors:** B. Farhadinia

**Affiliations:** Department of Mathematics, Quchan University of Advanced Technology, Quchan, Iran

## Abstract

The main goal of this contribution is to introduce the concept of higher order hesitant fuzzy soft set as an extension of fuzzy soft set that encompasses most of the existing extensions of fuzzy soft set as special cases. Furthermore, this new concept provides us with a method for dealing with multicriteria fuzzy decision making problems which are difficult to explain in other existing extensions of fuzzy soft set theory, especially when problems involve parameters with different-dimensional levels.

## 1. Introduction

Nowadays, many different extensions of FSs are known: L-fuzzy sets (L-FSs) [1], interval-valued fuzzy sets (IVFSs) [2], vague sets (VSs) [3], intuitionistic fuzzy sets (IFSs) [4], interval-valued intuitionistic fuzzy sets (IVIFSs) [5], linguistic fuzzy sets (LFSs) [6], type-2 fuzzy sets (T2FSs) [7], type-n fuzzy sets (TnFSs) [8], and fuzzy multisets (FMSs) [9].

An interesting extension of FSs is the hesitant fuzzy set (HFS) [10] in which the membership degree of an element is defined as a set of possible values. HFSs are quite suitable to deal with the situation where we have a set of possible values, rather than a margin of error (as in IFSs) or some possibility distribution on the possible values (as in T2FSs). Later, a number of other extensions of HFSs have been developed such as dual hesitant fuzzy sets (DHFSs) [11], generalized hesitant fuzzy sets (G-HFSs) [12], and hesitant fuzzy linguistic term sets (HFLTSs) [13].

However, HFS [10] and its recent generalization G-HFS [12] have their inherent drawbacks, because they express the membership degrees of an element to a given set only by crisp numbers or IFSs. In many practical decision making problems, the information provided by decision makers might often be described by FSs instead of crisp numbers or by other FS extensions instead of IFSs. This makes decision makers uncomfortable to provide exact crisp values or just IFSs for the membership degrees. Such a problem can be avoided by using the higher order HFS (HOHFS) introduced originally by the author in [14] for describing membership degrees. The HOHFS is fit for the situation where the decision makers have a hesitation among several possible memberships for an element. The HOHFS is the actual extension of HFS encompassing not only FSs, IFSs, VSs, T2FSs, FMSs, and HFSs, but also the recent extension of HFSs, called G-HFSs.

Since soft set theory was first initiated by Molodtsov in [15] as a new mathematical tool to deal efficiently with uncertainty and imprecision, many authors have become interested in applying this theory in various fields such as decision making [16], data analysis [17], and forecasting [18] recently. Moreover, a growing number of studies focus on the combination of FS and some extensions of FSs with soft set. Among them are, for instance, fuzzy soft set [19], intuitionistic fuzzy soft set [20], interval-valued fuzzy soft set [21], vague fuzzy soft set [22], and multifuzzy soft set [23].

The rationale behind this study is that on the one hand we tend to propose a more general extension of fuzzy soft set encompassing the existing ones, and on the other hand we try to model those situations where the existing kinds of fuzzy soft set are not applicable. We explain the rationale as follows.

As mentioned above and it will be discussed later, the HOHFS encompasses most of the fuzzy extensions as special cases, and therefore the combination of HOHFS with soft set contains most of the existing kinds of fuzzy soft set as special cases, too.

One of more interesting feature of the higher order hesitant fuzzy (HOHF) soft set which will be proposed in this contribution is that we can correspond each parameter to levels with different dimensions, the situation that cannot be modeled by the other kinds of fuzzy soft sets, specially by multifuzzy soft set. To make this clear, let us consider the situation discussed in [23]. Assume that *X* = {*x*_1_, *x*_2_, *x*_3_, *x*_4_, *x*_5_} is the universe set indicating the set of five kinds of colored drawing paper for engineering. Suppose the parameter set *A* = {*e*_1_, *e*_2_, *e*_3_} is a set of three criteria to evaluate the performance of these papers, where *e*_1_ stands for thickness which includes* three levels*: thick, average, and thin, *e*_2_ stands for color which consists of* three levels*: red, green, and blue, and *e*_3_ stands for ingredient which is made from* three levels*: cellulose, hemicellulose, and lignin. A company would like to select one of these papers depending on its performance. As discussed in [23], this decision problem can be analyzed using multifuzzy soft set. But if this decision problem is reconsidered slightly different from the original problem as *e*_1_ stands for thickness which includes* three levels*: thick, average, and thin, *e*_2_ stands for color which consists of* three levels*: red, green, and blue, and *e*_3_ stands for ingredient which is made from* two levels*: cellulose and hemicellulose where the level lignin is not considered, then, obviously, this situation cannot be handled not only by the existing kinds of fuzzy soft set theory, but also by multifuzzy soft set one in which all the parameters have to possess levels with the same dimension.

To deal with such cases, we should employ the concept of the HOHF soft set.

The present paper is organized as follows. An extension of HFSs, which is referred to as the higher order HFS (HOHFS), is introduced in Section 2.  Section 3 presents HOHF soft sets and discusses the properties of HOHF soft sets. In Section 4, we apply the proposed HOHF soft sets to multicriteria decision making problems involving parameters whose levels have different dimensions.  Section 5 presents an illustrative example. Finally, conclusion is drown in Section 6.

## 2. Preliminaries

This section is devoted to review the basic definitions and notions of fuzzy set (FS) and its new generalization which are originally referred to by Farhadinia [14] as the higher order hesitant fuzzy set (HOHFS). Actually, the HOHFS is a generalization of hesitant fuzzy set (HFS) introduced by Torra [10].

An ordinary fuzzy set (FS) *A* in *X* is defined [24] as *A* = {〈*x*, *A*(*x*)〉:*x* ∈ *X*}, where *A* : *X* → [0,1] and the real value *A*(*x*) represents the degree of membership of *x* in *A*.

**Definition 1 .**

Let *X* be the universe of discourse. A generalized type of fuzzy set (G-Type FS) on *X* is defined as

(1) $$\begin{array}{c} \tilde{A}=\left\{\langle x,\tilde{A}\left(x\right)\rangle :x\in X\right\}, \end{array}$$

where

(2) $$\begin{array}{c} \tilde{A}:X⟶\psi \left(\left[0,1\right]\right). \end{array}$$

Here, *ψ*([0,1]) denotes a family of crisp or fuzzy sets that can be defined within the universal set [0,1].

It is noteworthy that most of the existing extensions of ordinary FS are special cases of G-Type FS, for instance, [25]

(i) if *ψ*([0,1]) = [0,1], then the G-Type FS $\tilde{A}$ reduces to an ordinary FS;
(ii) if *ψ*([0,1]) = *ɛ*([0,1]) denoting the set of all closed intervals, then the G-Type FS $\tilde{A}$ reduces to an IVFS;
(iii) if *ψ*([0,1]) = *ℱ*([0,1]) denoting the set of all ordinary FSs, then the G-Type FS $\tilde{A}$ reduces to a T2FS;
(iv) if *ψ*([0,1]) = *L* denoting a partially ordered lattice, then the G-Type FS $\tilde{A}$ reduces to a L-FS.

**Definition 2 (see [26]).**

Let *X* be the universe of discourse. A hesitant fuzzy set (HFS) on *X* is symbolized by

(3) $$\begin{array}{c} H=\left\{\langle x,h\left(x\right)\rangle :x\in X\right\}, \end{array}$$

where *h*(*x*), referred to as the hesitant fuzzy element (HFE), is a set of some values in [0,1] denoting the possible membership degree of the element *x* ∈ *X* to the set *H*.

**Example 3 .**

If *X* = {*x*_1_, *x*_2_, *x*_3_} is the universe of discourse, *h*(*x*_1_) = {0.2,0.4,0.5}, *h*(*x*_2_) = {0.3,0.4}, and *h*(*x*_3_) = {0.3,0.2,0.5,0.6} are the HFEs of *x*_*i*_  (*i* = 1,2, 3) to a set *H*, respectively. Then *H* can be considered as a HFS; that is,

(4) H={〈x1,{0.2,0.4,0.5}〉,〈x2,{0.3,0.4}〉,iiiiiiii〈x3,{0.3,0.2,0.5.0.6}〉}.

As can be seen from Definition 2, HFS expresses the membership degrees of an element to a given set only by several real numbers between 0 and 1, while in many real-world situations assigning exact values to the membership degrees does not describe properly the imprecise or uncertain decision information. Thus, it seems to be difficult for the decision makers to rely on HFSs for expressing uncertainty of an element.

To overcome the difficulty associated with expressing uncertainty of an element to a given set, the concept of higher order hesitant fuzzy set (HOHFS) is introduced here to let the membership degrees of an element to a given set be expressed by several possible G-Type FSs.

**Definition 4 (see [14]).**

Let *X* be the universe of discourse. A higher order hesitant fuzzy set (HOHFS) on *X* is defined in terms of a function that when applied to *X* returns a set of G-Type FSs. A HOHFS is denoted by

(5) $$\begin{array}{c} \tilde{H}=\left\{\langle x,\tilde{h}\left(x\right)\rangle :x\in X\right\}, \end{array}$$

where $\tilde{h}(x)$, referred to as the higher order hesitant fuzzy element (HOHFE), is a set of some G-Type FSs denoting the possible membership degree of the element *x* ∈ *X* to the set $\tilde{H}$. In this regards, the HOHFS $\tilde{H}$ is also represented as

(6) $$\begin{array}{c} \tilde{H}=\left\{\langle x,\left\{{\tilde{h}}^{\left(1\right)}\left(x\right),\ldots ,{\tilde{h}}^{\left(l_{x}\right)}\left(x\right)\right\}\rangle :x\in X\right\}, \end{array}$$

where all ${\tilde{h}}^{\left(1\right)}(x),\ldots ,{\tilde{h}}^{\left(l_{x}\right)}(x)$ are G-Type FSs on *X*.

**Example 5 .**

If *X* = {*x*_1_, *x*_2_, *x*_3_} is the universe of discourse,

(7) iiih~(x1)={h~(1)(x1)=(0.2,0.4),h~(2)(x1)=(0.5,0.3)},iiih~(x2)={h~(1)(x2)=(0.3,0.4)},iiih~(x3)={h~(1)(x3)=(0.3,0.2),h~(2)(x3)=(0.1,0.3),iiiiiiiiiiiiiiiiih~(3)(x3)=(0.5,0.4)},

are the HOHFEs of *x*_*i*_(*i* = 1,2, 3) to a set $\tilde{H}$, respectively, where G-Type FSs ${\tilde{h}}^{\left(k\right)}(x_{i})=(\mu_{ki},\nu_{ki})$ are intuitionistic fuzzy set (IFS) [4] such that 0 ≤ *μ*_*ki*_, *ν*_*ki*_ ≤ 1 and 0 ≤ *μ*_*ki*_ + *ν*_*ki*_ ≤ 1 for *k* = 1,2,…, *l*_*x*_*i*__ and *i* = 1,2,…, |*X* | = 3. Then $\tilde{H}$ can be considered as a HOHFS; that is,

(8) H~={〈x1,{(0.2,0.4),(0.5,0.3)}〉,〈x2,{(0.3,0.4)}〉,iiiiiiii〈x3,{(0.3,0.2),(0.1,0.3),(0.5,0.4)}〉}.

In view of Definition 4, it is easily deduced that each HOHFS becomes a T2FS if all its G-Type FSs are the same and are in the form of T2FS. That is, if ${\tilde{h}}^{\left(1\right)}\left(x\right)=\cdots ={\tilde{h}}^{\left(l_{x}\right)}(x)=:\tilde{\bar{h}}(x)$ for any *x* ∈ *X*, then the HOHFS $\tilde{H}=\{\langle x,\tilde{\bar{h}}(x)\rangle :x\in X\}$ reduces to a T2FS.

It is noteworthy that the notions of interval-valued hesitant fuzzy set (IVHFS) [27] and interval type-2 fuzzy set (IT2FS) [28] both are special cases of HOHFSs. A HOHFS $\tilde{H}=\{\langle x,\tilde{h}(x)\rangle :x\in X\}$ reduces to an IVHFS, when all G-Type FSs ${\tilde{h}}^{\left(1\right)}(x),\cdots ,{\tilde{h}}^{\left(l_{x}\right)}(x)$ for any *x* ∈ *X* are considered as closed intervals of real numbers in [0,1]. Furthermore, an IVHFS $\tilde{H}=\{\langle x,[\tilde{h_{l}}\left(x\right),\tilde{h_{r}}\left(x\right)]\rangle :x\in X\}$ reduces to an IT2FS, when all intervals satisfy $\left[{\tilde{h}}_{l}^{\left(1\right)}\left(x\right),{\tilde{h}}_{r}^{\left(1\right)}\left(x\right)\right]=\cdots =[{\tilde{h}}_{l}^{\left(l_{x}\right)}(x),{\tilde{h}}_{r}^{\left(l_{x}\right)}(x)]=:[{\tilde{\bar{h}}}_{l}(x),{\tilde{\bar{h}}}_{r}(x)]$ for any *x* ∈ *X*.

Recently, HFSs are extended by intuitionistic fuzzy sets (IFSs) and referred to them as generalized hesitant fuzzy sets (G-HFSs) [12]. It is stated in [12] that FSs, IFSs, and HFSs are special cases of G-HFSs. Obviously, a G-HFS $\tilde{H}=\{\langle x,\tilde{h}(x)\rangle :x\in X\}$ is also an special case of HOHFS where all G-Type FSs ${\tilde{h}}^{\left(1\right)}(x),\ldots ,{\tilde{h}}^{\left(l_{x}\right)}(x)$ for any *x* ∈ *X* are considered as IFSs. This implies that HOHFSs are more useful than G-HFSs to deal with decision making, clustering, pattern recognition, image processing, and so forth, when experts have a hesitation among several possible memberships for an element to a set.

Having introduced HOHFEs of a HOHFS $\tilde{H}=\{\langle x,\tilde{h}(x)\rangle :x\in X\}$, we now turn our attention to the interpretation of HOHFS $\tilde{H}$ as the union of all HOHFEs; that is,

(9) $$\begin{array}{c} \tilde{H}=\underset{\tilde{h}\in \tilde{H}}{⋃}\left\{\tilde{h}\right\} \end{array}$$

which is of fundamental importance in the study of HOHFS aggregation and the higher order hesitant fuzzy soft matrix that will be introduced within the next parts of the paper.

**Definition 6 .**

Let ${\tilde{H}}_{1}={⋃}_{{\tilde{h}}_{1}\in {\tilde{H}}_{1}}\{\tilde{h_{1}}\}$ and ${\tilde{H}}_{2}={⋃}_{{\tilde{h}}_{2}\in {\tilde{H}}_{2}}\{\tilde{h_{2}}\}$ be two HOHFSs. We give the definition of operation ⊙ on HOHFSs based on its counterpart on HOHFEs as follows:

(10) H~1⊙H~2=⋃h1~∈H1~,h2~∈H2~{h1~⊙h2~}=⋃h1~∈H1~,h2~∈H2~{⋃hh1~∈h1~,hh2~∈h2~{hh1~⊙hh2~}},

where the last operation ⊙ stands for the operation on G-Type FSs.

Note that if there is no confusion, HOHFS operator, HOHFE operator, and G-Type FS operator are denoted by the same symbol.

Notice in the case that G-Type FSs in HOHFSs are reduced to crisp values, the G-Type FS operator in (10) is not the usual sum between real numbers because 0.6 + 0.5 = 1.1 ∉ [0,1], and indeed the G-Type FS operator is the* algebraic sum* where 0.6 ⊕ 0.5 = 0.6 + 0.5 − 0.6 × 0.5 = 0.3 ∈ [0,1]. Thus, the HOHFS operator is performed as the well-known HFS operator [29, 30].

**Theorem 7 .**

Let ${\tilde{h}}_{1}={⋃}_{{\tilde{h}}_{h1}\in {\tilde{h}}_{1}}\{\tilde{h_{h1}}\}$ and ${\tilde{h}}_{2}={⋃}_{{\tilde{h}}_{h2}\in {\tilde{h}}_{2}}\{\tilde{h_{h2}}\}$ be two HOHFEs. If the operation ⊙ on G-Type FSs has a property, denoted by $\tilde{\left(p_{G}\right)}$, then the operation ⊙ on HOHFEs has the counterpart of property $\tilde{\left(p_{G}\right)}$, denoted by $\tilde{\left(p\right)}$.

**Proof**

The proof is obvious from Definition 6. Suppose that, for example, the operation ⊙ on G-Type FSs is commutative; that is, for any G-Type FSs $\tilde{h_{h1}}\in \tilde{h_{1}},\tilde{h_{h2}}\in \tilde{h_{2}}$, the following equality holds:

(11) $$\begin{array}{c} \tilde{h_{h1}}⊙\tilde{h_{h2}}=\tilde{h_{h2}}⊙\tilde{h_{h1}}\;\;\left(\text{Property}\;\tilde{\left(p_{G}\right)}\right). \end{array}$$

Then, one can see from Definition 6 that

(12) h~1⊙h~2=⋃hh1~∈h1~,hh2~∈h2~{hh1~⊙hh2~}iiiiiiiiiii=⋃hh1~∈h1~,hh2~∈h2~{hh2~⊙hh1~}iiiiiiiiiii=h~2⊙h~1  (Property  (p)~).

This means that the operation ⊙ on HOHFEs is accordingly commutative.

By similar reasoning, one can easily prove that the HOHFE operator inherits all the properties of the G-Type FS operator.

As a corollary of Theorem 7, it can be observed that HOHFS operators inherit subsequently all operational properties of G-Type FS operators.

**Corollary 8 .**

Let ${\tilde{H}}_{1}={⋃}_{{\tilde{h}}_{1}\in {\tilde{H}}_{1}}\{\tilde{h_{1}}\}$ and ${\tilde{H}}_{2}={⋃}_{{\tilde{h}}_{2}\in {\tilde{H}}_{2}}\{\tilde{h_{2}}\}$ be two HOHFSs. If the operation ⊙ on HOHFEs has a property, denoted by $\tilde{\left(p\right)}$, then the operation ⊙ on HOHFSs has the counterpart of property $\tilde{\left(p\right)}$, denoted by $\tilde{\left(P\right)}$.

**Example 9 .**

Let ${\tilde{H}}_{1}={⋃}_{{\tilde{h}}_{1}\in {\tilde{H}}_{1}}\{\tilde{h_{1}}\}$ and ${\tilde{H}}_{2}={⋃}_{{\tilde{h}}_{2}\in {\tilde{H}}_{2}}\{\tilde{h_{2}}\}$ be two HOHFSs with HOHFEs in the form of IFSs. It can be shown for any IFSs $\tilde{h_{h1}},\tilde{h_{h2}}$ that

(13) iiiiihh1~⊕hh2~=hh2~⊕hh1~; (Property  (pG1)~),iiiiihh1~⊗hh2~=hh2~⊗hh1~; (Property  (pG2)~),λ(hh1~⊕hh2~)=λhh1~⊕λhh2~, λ>0;  (Property  (pG3)~),i(hh1~⊗hh2~)λ=hh1~λ⊗hh2~λ, λ>0;  (Property  (pG4)~),(λ1+λ2)hh1~=λ1hh1~⊕λ2hh1~,iiiiiiiiiiiiiiiiiiiiiiiiiiiiiiiiiiiiλ1,λ2>0;  (Property  (pG5)~),iiiiihh1~(λ1+λ2)=hh1~λ1⊗hh1~λ2,iiiiiiiiiiiiiiiiiiiiiiiiiiiiiiiiiλ1,λ2>0;  (Property  (pG6)~),

where (see e.g., [4])

(14) hh1~⊕hh2~=〈μhh1~,νhh1~〉⊕〈μhh2~,νhh2~〉iiiiiiiiiiiii=〈μhh1~+μhh2~−μhh1~·μhh2~,νhh1~·νhh2~〉;hh1~⊗hh2~=〈μhh1~,νhh1~〉⊗〈μhh2~,νhh2~〉iiiiiiiiiiiii=〈μhh1~·μhh2~,νhh1~+νhh2~−νhh1~·νhh2~〉;λhh1~=〈1−(1−μhh1~)λ,(νhh1~)λ〉, λ>0;hh1~λ=〈(μhh1~)λ,1−(1−νhh1~)λ〉, λ>0.

Accordingly, from Corollary 8, it is deduced that

(15) iiiiiiH1~⊕H2~=H2~⊕H1~; (Property  (P1)~),iiiiiiH1~⊗H2~=H2~⊗H1~; (Property  (P2)~),iλ(H1~⊕H2~)=λH1~⊕λH2~, λ>0;  (Property  (P3)~),i(H1~⊗H2~)λ=H1~λ⊗H2~λ, λ>0;  (Property  (P4)~),(λ1+λ2)H1~=λ1H1~⊕λ2H1~,iiiiiiiiiiiiiiiiiiiiiiiiiiiiiiiiiiiiiiiλ1,λ2>0;  (Property  (P5)~),iiiiH1~(λ1+λ2)=H1~λ1⊗H1~λ2,iiiiiiiiiiiiiiiiiiiiiiiiiiiiiiiiiiiiiiiλ1,λ2>0;  (Property  (P6)~).

## 3. HOHF Soft Sets and Their Properties

In the following, we first review briefly the concepts of soft set and fuzzy soft set. To do this, it may be helpful to recall that the sets *X*, *𝒫*(*X*), and *E* are referred to as the initial universe set, the power set of *X*, and a set of parameters, respectively.

**Definition 10 (see [15]).**

A pair (*F*, *A*) is called a soft set over *X*, where *F* is a mapping given by *F* : *A* ⊂ *E* → *𝒫*(*X*).

Indeed, a soft set over *X* is not a set but a parameterized family of subsets of *X*.

**Example 11 .**

Suppose that *X* = {*x*_1_, *x*_2_, *x*_3_, *x*_4_} is a set of houses under consideration and *A* = {*e*_1_, *e*_2_, *e*_3_} is a set of parameter, where *e*_1_ = expensive, *e*_2_ = beautiful, and *e*_3_ = in the good location. Let

(16) $$\begin{array}{c} F\left(e_{1}\right)=\left\{x_{1}\right\},\;F\left(e_{2}\right)=\left\{x_{1},x_{3}\right\},\;F\left(e_{3}\right)=\left\{x_{2},x_{4}\right\}. \end{array}$$

We use here the soft set (*F*, *A*) to describe the “attractiveness of the houses.” In this regard, *F*(*e*_1_) stands for “houses (expensive)” whose function value is the set {*x*_1_}, *F*(*e*_2_) means “houses (beautiful)” whose function value is the set {*x*_1_, *x*_3_}, and *F*(*e*_3_) implies “houses (in the good location)” whose function value is the set {*x*_2_, *x*_4_}.

**Definition 12 (see [19]).**

Let $\overset{˘}{F}:A\subset E\to \overset{˘}{𝒫}(X)$. A pair $\left(\overset{˘}{F},A\right)$ is called a fuzzy soft set over *X* where $\overset{˘}{𝒫}(X)$ is the set of all fuzzy subsets of *X*.

**Example 13 .**

Reconsider Example 11. We can describe the “attractiveness of the houses” under the fuzzy circumstances using the fuzzy soft set $\left(\overset{˘}{F},A\right)$, where

(17) F˘(e1)={x11,x20,x30,x40},F˘(e2)={x11,x20,x31,x40},F˘(e3)={x10,x21,x30,x41}.

This example shows that if we let the corresponding membership degrees of all elements *e* ∈ *E* − *A* be zero, then fuzzy soft sets $\left(\overset{˘}{F},A\right)$ and $\left(\overset{˘}{F},E\right)$ bear the same meaning and have the unified forms. This will bring the next discussions great convenience.

**Definition 14 .**

Let $\tilde{F}:E\to \tilde{𝒫}(X)$. A pair $\left(\tilde{F},E\right)$ is called a higher order hesitant fuzzy (HOHF) soft set over *X* where $\tilde{𝒫}(X)$ is the set of all higher order hesitant fuzzy subsets of *X*. In this regard, for any *e* ∈ *E*, we denote

(18) F~(e):={xh~F~(e)(x):x∈X}iiiiiiii={x{h~F~(e)(1)(x),…,h~F~(e)(lx)(x)}:x∈X},

where all ${\tilde{h}}_{\tilde{F}(e)}^{\left(1\right)}(x),\ldots ,{\tilde{h}}_{\tilde{F}(e)}^{\left(l_{x}\right)}(x)$ are G-Type FSs on *X*. That is to say, that the higher order hesitant fuzzy (HOHF) soft set over *X* can be denoted by

(19) $$\begin{array}{c} \left(\tilde{F},E\right)=\underset{x\in X}{\sum }\frac{x}{\left\{{\tilde{h}}_{\tilde{F}\left(e\right)}^{\left(1\right)}\left(x\right),\ldots ,{\tilde{h}}_{\tilde{F}\left(e\right)}^{\left(l_{x}\right)}\left(x\right)\right\}}. \end{array}$$

We hereafter denote all HOHF soft sets over *X* by HOHFS(*X*).

**Example 15 .**

Suppose that *X* = {*x*_1_, *x*_2_, *x*_3_, *x*_4_} is a set of color cloths under consideration and *A* = {*e*_1_, *e*_2_, *e*_3_} is a set of parameter where *e*_1_ =* color* which consists of red and blue, *e*_2_ =* ingredient* which is made from wool, cotton, and acrylic, and *e*_3_ =* price* which can be considered as high and low. In this situation, we should consider a higher order hesitant fuzzy soft set $\left(\tilde{F},E\right)$ as follows:

(20) F~(e1)={x1{(0.1,0.2),(0.3,0.2)},x2{(0.2,0.3),(0.3,0.1)},iiiiiiiix3{(0.3,0.3),(0.4,0.6)},x4{(0.1,0.3),(0.3,0.1)}},F~(e2)={x1{(0.5,0.2),(0.1,0.2),(0.3,0.2)},iiiiiiiiiiiiiix2{(0.2,0.4),(0.6,0.1),(0.3,0.1)},iiiiiiiiiiiiiix3{(0.3,0.3),(0.4,0.4),(0.4,0.6)},iiiiiiiiiiiiiix4{(0.1,0.3),(0.3,0.1),(0.2,0.5)}},F~(e3)={x1{(0.5,0,2),(0.4,0.1)},x2{(0.3,0.7),(0.6,0.1)},iiiiiiiix3{(0.4,0.4),(0.4,0.6)},x4{(0.8,0.2),(0.2,0.5)}},

where the above G-Type FSs, denoted by (*μ*, *ν*), are all in the form of IFS on *X*.

**Definition 16 .**

A higher order hesitant fuzzy soft set $\left(\tilde{F},E\right)$ over *X* is called a* null higher order hesitant fuzzy soft set* over *X* denoted by $\tilde{0}$, if for any *e* ∈ *E*

(21) $$\begin{array}{c} \tilde{F}\left(e\right):=\left\{\frac{x}{{\tilde{h}}_{\tilde{F}(e)}\left(x\right)}:x\in X\right\}=\left\{\frac{x}{\left\{\tilde{0},\ldots ,\tilde{0}\right\}}:x\in X\right\}, \end{array}$$

where $\tilde{0}$ is the zero G-Type FS on *X*.

**Definition 17 .**

A higher order hesitant fuzzy soft set $\left(\tilde{F},E\right)$ over *X* is called an* absolute higher order hesitant fuzzy soft set* over *X* denoted by $\tilde{1}$, if for any *e* ∈ *E*

(22) $$\begin{array}{c} \tilde{F}\left(e\right):=\left\{\frac{x}{{\tilde{h}}_{\tilde{F}(e)}\left(x\right)}:x\in X\right\}=\left\{\frac{x}{\left\{\tilde{1},\ldots ,\tilde{1}\right\}}:x\in X\right\}, \end{array}$$

where $\tilde{1}$ is the one G-Type FS on *X*.

**Definition 18 (see [14]).**

Consider two HOHFSs ${\tilde{H}}_{1}={⋃}_{{\tilde{h}}_{1}\in {\tilde{H}}_{1}}\{\tilde{h_{1}}\}$ and ${\tilde{H}}_{2}={⋃}_{{\tilde{h}}_{2}\in {\tilde{H}}_{2}}\{\tilde{h_{2}}\}$ on *X* = {*x*_1_, *x*_2_ …, *x*_*n*_}. The definition of distance measure for HOHFSs is given by

(23) $$\begin{array}{c} d\left({\tilde{H}}_{1},{\tilde{H}}_{2}\right)=\frac{1}{n}\overset{n}{\underset{i=1}{\sum }}d_{H}\left({\tilde{H}}_{1}\left(x_{i}\right),{\tilde{H}}_{2}\left(x_{i}\right)\right), \end{array}$$

where for each *x*_*i*_ ∈ *X*

(24) dH(H~1(xi),H~2(xi)) =max⁡⁡{max⁡h~1∈H~1min⁡h~2∈H~2D(h~1(xi),h~2(xi)),iiiiiiiiiiiiiiimin⁡h~1∈H~1max⁡h~2∈H~2D(h~1(xi),h~2(xi))}.

Here, *D*(·, ·) is a distance measure defined on G-Type FSs.

Notice that to have a correct comparison between two HOHFSs ${\tilde{H}}_{1}$ and ${\tilde{H}}_{2}$, the two following cases should be taken into account. The first case occurs if all G-Type FSs in each HOHFEs of HOHFS can be arranged increasingly (or decreasingly) by the use of a partial order. We refer to this case as* the ordered case*. Another case occurs when all G-Type FSs in each HOHFEs of HOHFS cannot be arranged increasingly (or decreasingly) using a partial order. This case is referred to as* the disordered case*.

In view of the above arguments, we shall represent the definition of a higher order hesitant fuzzy soft subset as follows.

**Definition 19 .**

Suppose that $\left(\tilde{F},E),(\tilde{G},E)\in \text{HOHFS}(X\right)$. $\left(\tilde{F},E\right)$ is said to be* a higher order hesitant fuzzy soft subset* of $\left(\tilde{G},E\right)$ if

(i) (in the ordered case) for all *e* ∈ *E* and for all *x* ∈ *X* it holds
  (25) $$\begin{array}{c} {\tilde{h}}_{\tilde{F}\left(e\right)}^{\left(k\right)}\left(x\right)⪯{\tilde{h}}_{\tilde{G}\left(e\right)}^{\left(k\right)}\left(x\right),\;\;1\leq k\leq l_{x},\; \end{array}$$
  where ⪯ stands for a partial order defined on G-Type FSs;
(ii) (in the disordered case) for all *e* ∈ *E* and for all *x* ∈ *X* one has
  (26) $$\begin{array}{c} d_{H}\left({\tilde{h}}_{\tilde{F}(e)}\left(x\right),\tilde{1}\right)\leq d_{H}\left({\tilde{h}}_{\tilde{G}(e)}\left(x\right),\tilde{1}\right), \end{array}$$
  where *d*_*H*_(·, ·) is that given in Definition 18 and $\tilde{1}$ is the absolute higher order hesitant fuzzy soft set over *X*.

The inclusion relation between $\left(\tilde{F},E\right)$ and $\left(\tilde{G},E\right)$ is denoted by $\left(\tilde{F},E)\tilde{⊑}(\tilde{G},E\right)$.

**Definition 20 .**

Two higher order hesitant fuzzy soft sets $\left(\tilde{F},E),(\tilde{G},E\right)$ are said to be* higher order hesitant fuzzy soft equal sets* if and only if $\left(\tilde{F},E)\tilde{⊑}(\tilde{G},E\right)$ and $\left(\tilde{G},E)\tilde{⊑}(\tilde{F},E\right)$.

**Definition 21 .**

Suppose that $\left(\tilde{F},E)\in \text{HOHFS}(X\right)$. $\left(\tilde{F^{c}},E\right)$ is said to be* a higher order hesitant fuzzy soft complementary set* of $\left(\tilde{F},E\right)$ if for all *e* ∈ *E* and for all *x* ∈ *X*

(27) $$\begin{array}{c} {\tilde{h}}_{\tilde{F^{c}}\left(e\right)}^{\left(k\right)}\left(x\right)={\left({\tilde{h}}_{\tilde{F}(e)}^{\left(k\right)}(x)\right)}^{c},\;1\leq k\leq l_{x}, \end{array}$$

where $({\tilde{h}}_{\tilde{F}(e)}^{\left(k\right)}(x){)}^{c}$ means the complement of the corresponding G-Type FSs ${\tilde{h}}_{\tilde{F}(e)}^{\left(k\right)}(x)$.

**Definition 22 .**

*Union* of $\left(\tilde{F},E),(\tilde{G},E)\in \text{HOHFS}(X\right)$, denoted by $\left(\tilde{F},E)\tilde{⊔}(\tilde{G},E\right)$, is defined for all *e* ∈ *E* as

(28) (M~,E) =(F~,E)⊔~(G~,E) =∑x∈Xx{(h~F~(e)(1)(x)∪h~G~(e)(1)(x)),…,(h~F~(e)(lx)(x)∪h~G~(e)(lx)(x))}.

**Definition 23 .**

*Intersection* of $\left(\tilde{F},E),(\tilde{G},E)\in \text{HOHFS}(X\right)$, denoted by $\left(\tilde{F},E)\tilde{⊓}(\tilde{G},E\right)$, is defined for all *e* ∈ *E* as

(29) (N~,E) =(F~,E)⊓~(G~,E) =∑x∈Xx{(h~F~(e)(1)(x)∩h~G~(e)(1)(x)),…,(h~F~(e)(lx)(x)∩h~G~(e)(lx)(x))}.

**Theorem 24 .**

Let $\left(\tilde{F},E),(\tilde{G},E)\in HOHFS(X\right)$. Then

(1) ${\left(\left(\tilde{F},E)\tilde{⊔}(\tilde{G},E\right)\right)}^{c}={\left(\tilde{F},E\right)}^{c}\tilde{⊓}{\left(\tilde{G},E\right)}^{c}:=\left(\tilde{F^{c}},E\right)\tilde{⊓}\left(\tilde{G^{c}},E\right)$,
(2) ${\left(\left(\tilde{F},E)\tilde{⊓}(\tilde{G},E\right)\right)}^{c}={\left(\tilde{F},E\right)}^{c}\tilde{⊔}{\left(\tilde{G},E\right)}^{c}:=(\tilde{F^{c}},E)\tilde{⊔}(\tilde{G^{c}},E)$.

**Proof**

* Part (*1*).* Assume that $\left(\tilde{M},E)=(\tilde{F},E)\tilde{⊔}(\tilde{G},E\right)$. In this regard, $\left((\tilde{F},E)\tilde{⊔}(\tilde{G},E){)}^{c}=(\tilde{M},E{)}^{c}:=(\tilde{M^{c}},E\right)$. On the other hand, from Definition 22 one gets

(30) ((F~,E)⊔~(G~,E))c =(Mc~,E) =∑x∈Xx{(h~F~(e)(1)(x)∪h~G~(e)(1)(x))c,…,(h~F~(e)(lx)(x)∪h~G~(e)(lx)(x))c} =∑x∈X(…,((h~F~(e)(lx)(x))c∩(h~G~(e)(lx)(x))c)})−1x(((h~F~(e)(lx)(x))c∩(h~G~(e)(lx)(x))c){((h~F~(e)(1)(x))c∩(h~G~(e)(1)(x))c),…,iiiiiiiiiiiiiiiiiiiiiiiii((h~F~(e)(lx)(x))c∩(h~G~(e)(lx)(x))c)})−1) =∑x∈Xx{(h~Fc~(e)(1)(x)∩h~Gc~(e)(1)(x)),…,(h~Fc~(e)(lx)(x)∩h~Gc~(e)(lx)(x))} =(Fc~,E)⊓~(Gc~,E) =(F~,E)c⊓~(G~,E)c    [Proved].

* Part (*2*).* The proof is similar to that of part (1).

**Theorem 25 .**

Let $\left(\tilde{F},E),(\tilde{G},E),(\tilde{K},E)\in \text{HOHFS}(X\right)$. Then

(1) $\left((\tilde{F},E)\tilde{⊔}(\tilde{G},E))\tilde{⊔}(\tilde{K},E)=(\tilde{F},E)\tilde{⊔}((\tilde{G},E)\tilde{⊔}(\tilde{K},E)\right)$,
(2) $\left((\tilde{F},E)\tilde{⊓}(\tilde{G},E))\tilde{⊓}(\tilde{K},E)=(\tilde{F},E)\tilde{⊓}((\tilde{G},E)\tilde{⊓}(\tilde{K},E)\right)$,
(3) $\left(\tilde{F},E\right)\tilde{⊔}\left(\left(\tilde{G},E\right)\tilde{⊓}\left(\tilde{K},E\right)\right)=\left(\left(\tilde{F},E\right)\tilde{⊔}\left(\tilde{G},E\right)\right)\tilde{⊓}\left(\left(\tilde{F},E\right)\tilde{⊔}\left(\tilde{K},E\right)\right)$,
(4) $\left(\tilde{F},E\right)\tilde{⊓}\left(\left(\tilde{G},E\right)\tilde{⊔}\left(\tilde{K},E\right)\right)=\left(\left(\tilde{F},E\right)\tilde{⊓}\left(\tilde{G},E\right)\right)\tilde{⊔}\left(\left(\tilde{F},E\right)\tilde{⊓}\left(\tilde{K},E\right)\right)$.

**Proof**

The proof is straightforward.

**Theorem 26 .**

Let $\left(\tilde{F},E)\in \text{HOHFS}(X\right)$, let $\tilde{1}$ be the absolute higher order hesitant fuzzy soft set over *X*, and let $\tilde{0}$ be the null higher order hesitant fuzzy soft set over *X*. Then

(1) $\left(\tilde{F},E)\tilde{⊔}(\tilde{F},E)=(\tilde{F},E\right)$,
(2) $\left(\tilde{F},E)\tilde{⊓}(\tilde{F},E)=(\tilde{F},E\right)$,
(3) $(\tilde{F},E)\tilde{⊔}\tilde{1}=\tilde{1}$,
(4) $\left(\tilde{F},E)\tilde{⊓}\tilde{1}=(\tilde{F},E\right)$,
(5) $(\tilde{F},E)\tilde{⊓}\tilde{0}=\tilde{0}$,
(6) $\left(\tilde{F},E)\tilde{⊔}\tilde{0}=(\tilde{F},E\right)$.

**Proof**

The proof is straightforward.

## 4. HOHF Soft Set Based Decision Making

In what follows, we are interested to present an application of HOHF soft set theory in a multiple criterion decision making problem. In order to solve decision making problems involving HOHF soft sets, an approach is proposed based on the concept of *λ*-level higher order hesitant fuzzy soft set.

**Definition 27 .**

Let $\left(\tilde{F},E)\in \text{HOHFS}(X\right)$ where |*X* | = *n* and |*E* | = *m*. The higher order hesitant fuzzy soft matrix $\tilde{𝔽}$ is defined in the concrete form of

(31) F~=(e1…emx1h~F~(e1)(x1)…h~F~(em)(x1)⋮⋮⋮xnh~F~(e1)(xn)…h~F~(em)(xn))ii=[h~F~(ej)(xi)]n×m.

We denote all higher order hesitant fuzzy soft matrix $\tilde{𝔽}$ over *X* by HOHFSM(*X*).

As can be seen from Definitions 27 and 14, there exists a bijective mapping from HOHFS(*X*) to HOHFS*M*(*X*), implying that any higher order hesitant fuzzy soft set $\left(\tilde{F},E)\in \text{HOHFS}(X\right)$ can be equivalent with its higher order hesitant fuzzy soft matrix $\tilde{𝔽}\in \text{HOHFSM}(X)$. This equivalent relationship allows us to identify any $\left(\tilde{F},E)\in \text{HOHFS}(X\right)$ with its $\tilde{𝔽}\in \text{HOHFS}M(X)$, interchangeably.

**Definition 28 .**

Let $\tilde{𝔽},\tilde{𝔾}\in \text{HOHFSM}(X)$. Some arithmetic operations on HOHFSM(*X*) can be defined as follows:

(32) F~⊕G~=[h~F~(ej)(xi)⊕h~G~(ej)(xi)]n×m,F~⊗G~=[h~F~(ej)(xi)⊗h~G~(ej)(xi)]n×m,iiiiκF~=[κ(h~F~(ej)(xi))]n×m, κ>0,iiiiF~κ=[(h~F~(ej)(xi))κ]n×m, κ>0,

where the right-hand-side operations are that considered on G-Type FSs in Definition 6.

Accordingly, from Definition 28 and Corollary 8, it can be deduced for any $\tilde{𝔽},\tilde{𝔾}\in \text{HOHFSM}(X)$ that

(33) iiiiiiiiF~⊕G~=G~⊕F~,iiiiiiiiF~⊗G~=G~⊗F~,iiiκ(F~⊕G~)=κF~⊕κG~, κ>0,iii(F~⊗G~)κ=F~κ⊗G~κ, κ>0,i(κ1+κ2)F~=κ1F~⊕κ2F~,   κ1,κ2>0,iiiiiiF~(κ1+κ2)=F~κ1⊗F~κ2, κ1,κ2>0.

The latter results can be employed whenever some experts are invited to conduct evaluation for one decision making problem. In such a situation, we are required to use aggregation operators on HOHFSM(*X*) given by experts to obtain a collective $\tilde{{𝔽}^{*}}\in \text{HOHFSM}(X)$. However, dealing with the aggregation methods based on multiexperts' higher order hesitant fuzzy soft matrix is beyond the scope of this paper.

Before introducing the concept of the *λ*-level higher order hesitant fuzzy soft set, we define a* threshold vector over parameter set E* as *λ*(*E*) = (*λ*(*e*_1_),…, *λ*(*e*_*m*_)) where $\lambda \left(e_{j}\right)={\tilde{h}}_{\lambda (e_{j})}\;(j=1,\ldots ,m)$. Obviously, the HOHFEs ${\tilde{h}}_{\lambda (e_{j})}\;(j=1,\ldots ,m)$ are changed dependently on the different parameter set *E*.

Consider again the higher order hesitant fuzzy soft matrix $\tilde{𝔽}$ given in the concrete form of $\tilde{𝔽}=[{\tilde{h}}_{\tilde{F}(e_{j})}(x_{i}){]}_{n\times m}$. In order to get reasonably a HOHFE for each parameter *e*_*j*_ ∈ *E* from $\tilde{𝔽}$, we can employ some aggregation operators.

We define

(34) 〈h~λ(ej)〉Agg=Agg(h~F~(ej)(x1),…,h~F~(ej)(xn)),iiiiiiiiiiiiiiiiiiiiiiiiiiiiiiiiiiiiiiej∈E(j=1,…,m),

where Agg(·) is an aggregation operator on HOHFEs.

In view of the latter setting, the threshold vector over parameter set *E* is defined as

(35) λAgg(E)={λAgg(ej), j=1,…,m}iiiiiiiiiiii={〈h~λ(ej)〉Agg, j=1,…,m}.

**Definition 29 .**

A real function *I* : HOHFE(*X*) × HOHFE(*X*)→[0,1] is called the inclusion measure of the HOHFEs ${\tilde{h}}_{\tilde{F}(e)}(x)=\{{\tilde{h}}_{\tilde{F}(e)}^{\left(k\right)}(x){\}}_{k=1}^{l_{x}}$, ${\tilde{h}}_{\tilde{G}(e)}(x)=\{{\tilde{h}}_{\tilde{G}(e)}^{\left(k\right)}(x){\}}_{k=1}^{l_{x}}$, and ${\tilde{h}}_{\tilde{K}(e)}(x)=\{{\tilde{h}}_{\tilde{K}(e)}^{\left(k\right)}(x){\}}_{k=1}^{l_{x}}$, if *I*(·, ·) satisfies the following properties.

(1) $I(\tilde{1},\tilde{0})=0$.
(2) $I({\tilde{h}}_{\tilde{F}(e)}(x),{\tilde{h}}_{\tilde{G}(e)}(x))=1$ iff
  (36) h~F~(e)(x)⊆~h~G~(e)(x) iff    h~F~(e)(k)(x)⊆h~G~(e)(k)(x),iiiiiiiiiiiiiiiiiiiiiiiiiiiiiiiiiiiiiiiiiiiik=1,…,lx,
 where the last inclusion operator ⊆ is that introduced on G-Type FSs.
(3) If ${\tilde{h}}_{\tilde{F}(e)}(x)\tilde{\subseteq }{\tilde{h}}_{\tilde{G}(e)}(x)\tilde{\subseteq }{\tilde{h}}_{\tilde{K}(e)}(x)$, then $I({\tilde{h}}_{\tilde{K}(e)}(x),{\tilde{h}}_{\tilde{F}(e)}(x))\leq I({\tilde{h}}_{\tilde{G}(e)}(x),{\tilde{h}}_{\tilde{F}(e)}(x))$ and $I({\tilde{h}}_{\tilde{K}(e)}(x),{\tilde{h}}_{\tilde{F}(e)}(x))\leq I({\tilde{h}}_{\tilde{K}(e)}(x),{\tilde{h}}_{\tilde{G}(e)}(x))$.

Let us now define the following two-valued function *i*(·, ·) on G-Type FSs ${\tilde{h}}_{\tilde{F}(e)}^{\left(k\right)}(x)$ and ${\tilde{h}}_{\tilde{G}(e)}^{\left(k\right)}(x)$ as

(37) i(h~F~(e)(k)(x),h~G~(e)(k)(x)) ={1,if  h~F~(e)(k)(x)⊆  h~G~(e)(k)(x),    k=1,…,lx,;0,otherwise,

and then, the following function is an inclusion measure of the HOHFEs ${\tilde{h}}_{\tilde{F}(e)}(x)=\{{\tilde{h}}_{\tilde{F}(e)}^{\left(k\right)}(x){\}}_{k=1}^{l_{x}}$ and ${\tilde{h}}_{\tilde{G}(e)}(x)=\{{\tilde{h}}_{\tilde{G}(e)}^{\left(k\right)}(x){\}}_{k=1}^{l_{x}}$ given by

(38) $$\begin{array}{c} I\left({\tilde{h}}_{\tilde{F}(e)}\left(x\right),{\tilde{h}}_{\tilde{G}(e)}\left(x\right)\right)=\frac{1}{l_{x}}\overset{l_{x}}{\underset{k=1}{\sum }}i\left({\tilde{h}}_{\tilde{F}\left(e\right)}^{\left(k\right)}\left(x\right),{\tilde{h}}_{\tilde{G}\left(e\right)}^{\left(k\right)}\left(x\right)\right). \end{array}$$

**Definition 30 .**

We define the *λ*(*E*)-*level higher order hesitant fuzzy soft set* of $\tilde{𝔽}\in \text{HOHFSM}(X)$, denoted by $L(\tilde{𝔽},\lambda (E))$, as follows:

(39) L(F~,λ(ej))={xi∈X ∣ I(λ(ej),h~F~(ej)(xi))>0},iiiiiiiiiiiiiiiiiiiiiiiiiiiiiiiiiiiiiiiiiii∀ej∈E(j=1,…,m),

where *I*(·, ·) is the inclusion measure introduced by (38).

As a result from Definition 30 and (35), the *λ*_Agg_(*E*)-level higher order hesitant fuzzy soft set of $\tilde{𝔽}\in \text{HOHFSM}(X)$ is characterized by

(40) L(F~,λAgg(ej)) ={xi∈X ∣ I(〈h~λ(ej)〉Agg,h~F~(ej)(xi))>0}.

Once the *λ*(*E*)-level higher order hesitant fuzzy soft set of $\tilde{𝔽}$ is achieved, we calculate the inclusion measure values $I(\lambda (e_{j}),{\tilde{h}}_{\tilde{F}(e_{j})}(x_{i}))$ for *e*_*j*_  (*j* = 1,…, *m*) and *x*_*i*_  (*i* = 1,…, *n*) given by (38) to rank all the alternatives. The optimal decision is then selected as *x*_*k*_ if

(41) ∑j=1mI(λ(ej),h~F~(ej)(xk)) =max⁡i=1,…,n{∑j=1mI(λ(ej),h~F~(ej)(xi))}.

It is noteworthy to say that if HOHFEs ${\tilde{h}}_{\tilde{F}(e_{j})}(x_{i})$ reduce to IFSs, then the inclusion measure values $I(\lambda (e_{j}),{\tilde{h}}_{\tilde{F}(e_{j})}(x_{i}))$ are coincided to choice values *C*_*i*_ of *x*_*i*_  (*i* = 1,…, *n*) introduced in [31].

Now we are in a position to present an algorithm for solving higher order hesitant fuzzy soft set based decision making problem by using *λ*(*E*)-level higher order hesitant fuzzy soft sets initiated in this study.

**Algorithm 31 .**

Consider the following.

**Step 1 .**

Input the higher order hesitant fuzzy soft matrix $\tilde{𝔽}$ over a finite initial universe *X* and a finite parameter set *E*.

**Step 2 .**

Compute the threshold vector *λ*(*E*) = (*λ*(*e*_1_),…, *λ*(*e*_*m*_)) corresponding to $\tilde{𝔽}$.

**Step 3 .**

Calculate inclusion measure value of all alternatives to determine their ranking and then selecting the optimal decision by the use of (41).

## 5. Illustrative Example

To illustrate the idea of Algorithm 31, let us take into consideration the following example which addresses Example 15 with more details.

**Example 32 .**

Suppose that *X* = {*x*_1_, *x*_2_, *x*_3_, *x*_4_} is a set of color cloths under consideration and *A* = {*e*_1_, *e*_2_, *e*_3_} is a set of parameter where *e*_1_ =* color* which consists of red and blue, *e*_2_ =* ingredient* which is made from wool, cotton, and acrylic, and *e*_3_ =* price* which can be considered as high and low. In this situation, we should consider a higher order hesitant fuzzy soft set $\left(\tilde{F},E\right)$ as follows:

(42) F~(e1)={x1{(0.1,0.2),(0.3,0.2)},x2{(0.2,0.3),(0.3,0.1)},iiiiiiiiiiiiix3{(0.3,0.3),(0.4,0.6)},x4{(0.1,0.3),(0.3,0.1)}},F~(e2)={x1{(0.5,0.2),(0.1,0.2),(0.3,0.2)},iiiiiiiiiiiiix2{(0.2,0.4),(0.6,0.1),(0.3,0.1)},iiiiiiiiiiiiix3{(0.3,0.3),(0.4,0.4),(0.4,0.6)},iiiiiiiiiiiiix4{(0.1,0.3),(0.3,0.1),(0.2,0.5)}},F~(e3)={x1{(0.5,0,2),(0.4,0.1)},x2{(0.3,0.7),(0.6,0.1)},iiiiiiiiiiiiix3{(0.4,0.4),(0.4,0.6)},x4{(0.8,0.2),(0.2,0.5)}},

where the above G-Type FSs, denoted by (*μ*, *ν*), are all in the form of IFS on *X*.

The aim here is to select one of color cloths depending on its performance evaluated with respect to all parameters.

The tabular representation of the above higher order hesitant fuzzy soft matrix $\tilde{𝔽}$ is given in Table 1.

To perform the second step of Algorithm 31, we need to compute a proper threshold vector over parameter set *E*. First of all we evaluate the efficiency of two well-known aggregation operators which are called the arithmetic average operator (AAO) and the geometric average operator (GAO) [32]. Based on the aggregation AAO, the threshold vector over parameter set *E* is defined as

(43) λAAO(E)={λAAO(ej), j=1,…,m}iiiiiiiiiiii={〈h~λ(ej)〉AAO, j=1,…,m},

where

(44) 〈h~λ(ej)〉AAO=1n⊕i=1nh~F~(ej)(xi)iiiiiiiiiiiiiiiii={1n⊕i=1nh~F~(ej)(1)(xi),…,1n⊕i=1nh~F~(ej)(lx)(xi)}.

Needless to say that the operator ⊕ on ${\tilde{h}}_{\tilde{F}\left(e_{j}\right)}^{\left(k\right)}\left(x_{i}\right)\;(k=1,\ldots ,l_{x})$ is that defined on G-Type FSs.

In this example, operator ⊕ considered on IFSs is given for IFSs ${\tilde{h}}_{\tilde{F}\left(e_{j}\right)}^{\left(k\right)}\left(x_{i}\right)=\left(\mu_{i}^{k,j},\nu_{i}^{k,j}\right),\;i=1,\ldots ,n$ by [33]

(45) 1n⊕i=1nh~F~(ej)(k)(xi):=(μk,j,νk,j)iiiiiiiiiiiiiiiiiiiiiiII=(1−∏i=1n(1−μik,j),∏i=1nνik,j).

Furthermore, based on the aggregation GAO, we have

(46) 〈h~λ(ej)〉GAO =(⊗i=1nh~F~(ej)(xi))1/n ={(⊗i=1nh~F~(ej)(1)(xi))1/n,…,(⊗i=1nh~F~(ej)(lx)(xi))1/n  },

where for IFSs ${\tilde{h}}_{\tilde{F}\left(e_{j}\right)}^{\left(k\right)}\left(x_{i}\right)=\left(\mu_{i}^{k,j},\nu_{i}^{k,j}\right),\;i=1,\ldots ,n$, we define

(47) (⊗i=1nh~F~(ej)(k)(xi))1/n:=(μk,j,νk,j)iiiiiiiiiiiiiiiiiiiiiiiiiiiiii=(∏i=1nμik,j,1−∏i=1n(1−νik,j)).

**Proposition 33 .**

Assume that

(48) 〈h~λ(ej)(k)〉 AAO =1n⊕i=1nh~F~(ej)(k)(xi):=(μk,j,νk,j)iiiiiiiiiiiiiiiiiiiiiiiiiiiiiiiiiiiiii=(1−∏i=1n(1−μik,j),∏i=1nνik,j),〈h~λ(ej)(k)〉 GAO =(⊗i=1nh~F~(ej)(k)(xi))1/n:=(μk,j,νk,j)iiiiiiiiiiiiiiiiiiiiiiiiiiiiiiiiiiiiii=(∏i=1nμik,j,1−∏i=1n(1−νik,j)).

Then, for any *x*_*i*_ ∈ *X*

(49) I(〈h~λ(ej)〉 AAO ,h~F~(ej)(xi))=0,I(〈h~λ(ej)〉 GAO ,h~F~(ej)(xi))=1,

where *I*(·, ·) is the inclusion measure given by (38).

**Proof**

The proof is straightforward from the fact that for any indices *i*, *j*, *k*

(50) (μik,j,νik,j)⊆(1−∏i=1n(1−μik,j),∏i=1nνik,j),(μik,j,νik,j)⊇(∏i=1nμik,j,1−∏i=1n(1−νik,j)).

The above proposition demonstrates that the threshold vectors over parameter set *E* defined in accordance with the aggregations AAO and GAO are not efficient, because indifferent inclusion measure values for the whole alternatives *x*_*i*_ ∈ *X* do not allow us to have a promising judgment about optimal decision.

In order to overcome adverse effect of indifferent inclusion measure values, we use, but we are not limited to, median operator [20] instead of AAO and GAO. The threshold vector based on median operator can be computed as follows.

Let us denote any IFS ${\tilde{h}}_{\tilde{F}(e_{j})}^{\left(k\right)}(x_{i})$ by ${\tilde{h}}_{\tilde{F}(e_{j})}^{\left(k\right)}(x_{i})=(\mu_{i}^{k,j},\nu_{i}^{k,j})$ for 1 ≤ *i* ≤ *n*, 1 ≤ *j* ≤ *m*, 1 ≤ *k* ≤ *l*_*x*_. Suppose that membership degree (resp., nonmembership degree) of all alternatives in *X* is arranged in increasing order, and then *x*_*σ*(*i*)_ is referred to as the alternative with *i*th largest membership degree (resp., nonmembership degree). In this regards, we define

(51) $$\begin{array}{c} {\langle {\tilde{h}}_{\lambda \left(e_{j}\right)}^{\left(k\right)}\rangle }_{\text{med}}:=\left(\mu_{\text{med}}^{k,j},\nu_{\text{med}}^{k,j}\right), \end{array}$$

where

(52) μmedk,j={μσ((m+1)/2)k,j,if  m  is  odd;12(μσ(m/2)k,j+μσ(m/2+1)k,j),if  m  is  even,νmedk,j={νσ((m+1)/2)k,j,  if  m  is  odd;  12(νσ(m/2)k,j+νσ(m/2+1)k,j),  if  m  is  even.  

Now, the threshold vector *λ*_med_(*E*) can be obtained as represented in tabular form in Table 2.

By applying the third step of Algorithm 31, we find that the optimal decision is to select *x*_2_. The results obtained through Step 3 of Algorithm 31 are summarized in Table 3.

## 6. Conclusion

This paper proposed a more general extension of fuzzy soft set that not only encompasses the existing ones as special cases, but also can be applied to model those situations where the existing kinds of fuzzy soft set are not applicable. The proposed higher order hesitant fuzzy soft set provides us with a multicriteria decision making method to deal with problems involving parameters with different-dimensional levels. As future work, we consider the study of aggregation methods based on multiexperts' higher order hesitant fuzzy soft matrix where some experts are invited to conduct evaluation for one decision making problem.

## Figures and Tables

**Table 1:** Higher order hesitant fuzzy soft matrix $\tilde{𝔽}$.

	*e*_1_	*e*_2_	*e*_3_
*x*_1_	{(0.1,0.2), (0.3,0.2)}	{(0.5,0.2), (0.1,0.2), (0.3,0.2)}	{(0.5,0, 2), (0.4,0.1)}
*x*_2_	{(0.2,0.3), (0.3,0.1)}	{(0.2,0.4), (0.6,0.1), (0.3,0.1)}	{(0.3,0.7), (0.6,0.1)}
*x*_3_	{(0.3,0.3), (0.4,0.6)}	{(0.3,0.3), (0.4,0.4), (0.4,0.6)}	{(0.4,0.4), (0.4,0.6)}
*x*_4_	{(0.1,0.3), (0.3,0.1)}	{(0.1,0.3), (0.3,0.1), (0.2,0.5)}	{(0.8,0.2), (0.2,0.5)}

**Table 2:** Higher order hesitant fuzzy soft matrix $\tilde{𝔽}$ and the threshold vector λ_med_(*E*).

	*e*_1_	*e*_2_	*e*_3_
*x*_1_	{(0.1,0.2), (0.3,0.2)}	{(0.5,0.2),(0.1, 0.2), (0.3,0.2)}	{(0.5,0, 2), (0.4,0.1)}
*x*_2_	{(0.2,0.3), (0.3,0.1)}	{(0.2,0.4), (0.6,0.1), (0.3,0.1)}	{(0.3,0.7), (0.6,0.1)}
*x*_3_	{(0.3,0.3), (0.4,0.6)}	{(0.3,0.3), (0.4,0.4), (0.4,0.6)}	{(0.4,0.4), (0.4,0.6)}
*x*_4_	{(0.1,0.3), (0.3,0.1)}	{(0.1,0.3), (0.3,0.1), (0.2,0.5)}	{(0.8,0.2), (0.2,0.5)}
$\langle {\tilde{h}}_{\lambda (e)}\rangle_{{}_{\text{med}}}$	{(0.15,0.3), (0.3,0.15)}	{(0.25,0.3), (0.35,0.15), {(0.3,0.3)}	{(0.35,0.3), (0.4,0.3)}

**Table 3:** Inclusion measure values corresponding to λ_med_(*E*).

	*e*_1_	*e*_2_	*e*_3_	Total
*x*_1_	0	2/3	1	5/3
*x*_2_	1	2/3	1/2	13/6
*x*_3_	1/2	1/3	0	5/6
*x*_4_	1/2	0	1/2	1
